# Identification of Immune Cell Landscape and Construction of a Novel Diagnostic Nomogram for Crohn’s Disease

**DOI:** 10.3389/fgene.2020.00423

**Published:** 2020-04-29

**Authors:** Hong Chen, Chunqiu Chen, Xiaoqi Yuan, Weiwei Xu, Mu-qing Yang, Qiwei Li, Zhenyu Shen, Lu Yin

**Affiliations:** Center for Difficult and Complicated Abdominal Surgery, Shanghai Tenth People’s Hospital, Tongji University School of Medicine, Shanghai, China

**Keywords:** inflammatory bowel diseases, Crohn’s disease, CIBERSORTx, immune cells, GSVA, nomogram

## Abstract

Crohn’s disease (CD) has an increasing incidence and prevalence worldwide. The etiology of CD remains unclear and there is no gold standard for diagnosis. The dysregulated immune response and different infiltration status of immune cells are critical for CD pathogenesis; therefore, it is important to provide an overview of immune-cell alterations in CD and explore a novel method for auxiliary diagnosis. Here we analyzed microarray datasets from Gene Expression Omnibus (GEO), and an extended version of Cell-type Identification By Estimating Relative Subsets Of RNA Transcripts (CIBERSORTx) was utilized to estimate the fraction of 22 types of immune cells. Differentially expressed genes (DEGs) and a protein-protein interaction (PPI) network were identified, and we performed gene set enrichment analysis (GSEA) and gene set variation analysis (GSVA) to identify differentially regulated pathways in CD. Least absolute shrinkage and selection operator (LASSO) regression was conducted to filter features, and a diagnostic nomogram based on logistic regression was built and validated in an independent validation cohort. In the derivation cohort, we found a proportion of 17 immune-cell types to be significantly altered between CD and healthy controls and a total of 150 DEGs were identified, which were mostly related to the immune response. Among the 15 hub genes based on the PPI network, C-X-C chemokine ligand 8 (CXCL8) and interleukin-1B (IL-1B) showed the highest degree of interaction. Additionally, GSEA and GSVA identified five significantly enriched pathways, among which the nucleotide-binding oligomerization domain (NOD)-like receptor signaling pathway was critical in the CD development. Furthermore, six variables comprising of CXCL8, IL-1B, M1 macrophages, regulatory T cells, CD8^+^ T cells, and plasma cells were identified by LASSO regression and incorporated into a logistic regression model. The nomogram displayed a good prediction, with a 0.915 area under the receiver operating curve (AUC) and a C-index of 0.915 [95% confidence interval (CI): 0.875–0.955]. Similar results were found in the validation cohort, with an AUC of 0.884 and a 0.884 C-index (95% CI: 0.843–0.924). These results provide novel *in silico* insight into cellular and molecular characteristics of CD and potential biomarkers for diagnosis and targeted therapy.

## Introduction

Inflammatory bowel diseases (IBDs) are disorders characterized by chronic inflammation that affects the digestive tract and includes the following two main components: ulcerative colitis (UC) and Crohn’s disease (CD). CD can cause segmental and transmural damage in any section of the entire digestive tract, especially in the distal ileum and colon (Feuerstein and Cheifetz, 2017). There is currently no single gold standard for CD diagnosis; therefore a combination of endoscopic examination, histology, and clinical manifestation are recommended (Gomollon et al., 2017). However, in most cases, the clinical significance of CD histological hallmarks is low, and no medical treatments can cure CD completely (Feakins, 2013). Therefore, it is important to clarify the cellular and molecular mechanisms associated with CD pathogenesis and find novel intervention targets and explore potential biomarkers as diagnostic and prognostic indicators.

Numerous investigations of CD pathogenesis have focused on the interplay between environmental factors, genetics, gut microflora, and immune responses (Torres et al., 2017). As hallmarks of CD, the roles of immune cells in dysregulated intestinal immune responses are crucial and remain to be further elucidated (de Souza and Fiocchi, 2016). For example, T cells can cause mucosal damage in gut tissues by producing inflammatory cytokines, a process that can be blocked by treatment with the anti-α4 integrin antibody natalizumab (Neurath, 2014, 2017). However, different subpopulations of T cells can differentially contributes (Smids et al., 2018). Traditional approaches for assessing tissue composition, such as immunohistochemistry and flow cytometry, are limited due to the difficulty in simultaneous identification of multiple immune-cell types, as well as their low throughput. Therefore, a comprehensive assessment of the heterogeneity of immune cells in CD occurrence is important.

With the development of microarray and high-throughput sequencing technologies, estimation of cell proportions from bulk tissues can be performed using genomics data via bioinformatics techniques (Shen-Orr and Gaujoux, 2013). Cell-type Identification By Estimating Relative Subsets Of RNA Transcripts (CIBERSORT) is a deconvolution algorithm that can analyze 22 distinct immune-cell subsets in complex tissues based on normalized bulk transcriptome profiles (Newman et al., 2015)and has been successfully used in different types of cancer (Peng et al., 2019; Yang et al., 2019; Zhang et al., 2019), as well as non-tumor diseases such as idiopathic pulmonary fibrosis and systemic lupus erythematosus (Liu et al., 2018; Panousis et al., 2019). In 2019, the developer of CIBERSORT introduced an updated version (CIBERSORTx) that provides more accurate portraits of tissue composition based on cell-expression signatures via single-cell experiments (Newman et al., 2019). These significant advances of the deconvolution algorithm provide the probability to comprehensively characterize infiltrating immune cells in CD.

In the present study, we performed a comprehensive analysis by integrating two expression profiling microarray datasets and exploring the proportion of intestinal immune-cell types for each individual using CIBERSORTx. After identifying differentially expressed genes (DEGs), we performed gene set enrichment analysis (GSEA) and gene set variation analysis (GSVA) to find signaling pathways involved in CD occurrence. We then constructed a multivariable logistic regression model based on the key features of this information and built a nomogram to provide a novel method for CD auxiliary diagnosis. Furthermore, an external cohort comprising of four microarray datasets was used for independent validation of the nomogram. The results provide in-depth insights into the cellular and molecular mechanisms related to clinical management of CD.

## Materials and Methods

### Microarrays Datasets Collection

The raw data of microarray datasets [GSE112366 (VanDussen et al., 2018) and GSE75214 (Vancamelbeke et al., 2017)] were downloaded from the NCBI Gene Expression Omnibus (GEO) database (Barrett et al., 2013) and merged as a derivation cohort. The two datasets were based on the platforms of GPL13158 [(HT_HG-U133_Plus_PM) Affymetrix HT HG-U133 + PM Array Plate] and GPL6244 {[HuGene-1_0-st] Affymetrix Human Gene 1.0 ST Array [transcript (gene) version]}, respectively. The dataset GSE112366 contains 141 CD samples without any treatment and 26 normal samples from the ileum. Another dataset (GSE75214) contains 75 CD samples and 22 normal samples obtained from the ileum or colon.

### Data Preprocessing and CIBERSORTx Estimation

Each expression matrix was extracted from the raw data using R package “affy” (Gautier et al., 2004) and then normalized and transformed into a log_2_-based logarithm by robust multi-array average algorithm (Irizarry et al., 2003). After matrix merging, the R package “sva” (Leek et al., 2012) and the function “combat” were used to remove the batch effects and other unnecessary variations. CIBERSORTx was employed to determine the proportion of each immune cell involved in CD patients and healthy individuals. The gene expression data was uploaded to the CIBERSORTx web portal^1^, and the algorithm was run using the LM22 signature for 100 permutations. The LM22 signature matrix defined 22 infiltrating immune-cell components, including subsets of macrophages, T cells, natural killer (NK) cells, mast cells, B cells, dendritic cells (DC), monocytes, plasma cells, neutrophils, and eosinophils (Newman et al., 2015). We used bulk-mode batch correction, and the output was in absolute mode according to the tutorial on the web site and reflects the absolute proportion of each cell type in the mixture. Only cases with a CIBERSORTx output *P* < 0.05 were chosen for further analysis. The Wilcoxon test was used to analyze differences in immune cell fractions between CD patients and healthy controls.

### Identification of DEGs and Functional Enrichment Analyses

The R package “limma” (Ritchie et al., 2015) was used to perform DEG analysis by comparing CD and healthy control groups. For DEG identification, the cut-off criteria of | log_2_FC| > 1 and adjusted *P* < 0.05 were regard as statistically significant. The biological function of DEGs was identified by Gene Ontology (GO) and Kyoto Encyclopedia of Genes and Genomes (KEGG) pathway enrichment analyses by using the R package “clusterProfiler” (Yu et al., 2012). Fisher’s exact test was employed, and the occurrence of false positives was corrected by Benjamini-Hochberg (B-H) multiple test correction method. An adjusted *P* < 0.05 was set as the cut-off criterion. The Search Tool for the Retrieval of Interacting Genes (STRING) online database^2^ was used to construct the protein-protein interaction (PPI) networks for the DEGs (Szklarczyk et al., 2019), with an interaction score >0.4 was regarded as statistically significant. Subsequently, the molecular interaction network was visualized using Cytoscape software (v 3.7.1) (Shannon et al., 2003). Furthermore, we used the Cytohubba plugin app within Cytoscape to calculate degree of interaction between DEGs and defined the top 15 genes as hub genes (Chin et al., 2014).

### GSEA and GSVA Analysis

GSEA is a calculation method to explore whether *a priori* defined genomes between two groups show significant differences. Therefore, we used GSEA software^3^ to evaluate the differentially enriched pathways between CD patients and healthy controls (Subramanian et al., 2005). The previously annotated gene set c2.cp.kegg.v6.2.symbols.gmt was chosen as the reference gene list. The results with a cut-off criterion of a nominal *P* < 0.05 were considered statistically significant. Furthermore, we performed GSVA to assess the underlying changes in pathway activity. This method is a non-parametric unsupervised method that transforms the genes of the sample matrix into predefined gene sets without *a priori* knowledge of experiment design (Hanzelmann et al., 2013). In the present study, we used the R package “GSVA” to calculate the scores for each patient based on previously defined gene sets of KEGG pathways. Subsequently, the R package limma was used to build linear models for comparing GSVA scores between CD patients and healthy controls and we defined pathways with a *P* < 0.05 and | log_2_FC| ≥ 0.2 as significantly altered.

### Co-expression Analysis and Construction of Predictive Nomogram

To elucidate interaction between immune cells, hub genes, and pathways in CD, we indentified co-expression patterns based on Spearman correlation analysis. To identify the immune cells critical to CD progression, we established criteria including a correlation coefficient >0.3 and a *P* < 0.05 and incorporated the immune cells and related hub genes and pathways into the least absolute shrinkage and selection operator (LASSO) regression model in order to select the optimal predictive features.

A multivariable logistic regression model and a nomogram were constructed by integrating the features with non-zero coefficients in LASSO regressionin in order to prevent model overfitting. Model sensitivity and specificity were evaluated by receiver operating characteristic (ROC) analyses. The calibration of the nomogram was assessed by comparing the observed actual diagnosis with the predicted probability by plotting a calibration curve. The C-index was measured to quantify the discriminative performance of the nomogram. The Pearson residuals plot was used to assess whether the model is fitted properly (Zhang, 2016).

### Validation of the Nomogram

The diagnostic nomogram was applied to an independent cohort comprising microarray datasets from the GEO [GSE3365 (Burczynski et al., 2006), GSE10616 (Kugathasan et al., 2008), GSE16879 (Arijs et al., 2009), and GSE102133 (Verstockt et al., 2019)] to evaluate the efficacy of model validation and prediction. These four datasets were based on the platforms GPL96 [(HG-U133A) Affymetrix Human Genome U133A Array], GPL5760 (Affymetrix GeneChip Human Genome U133 Plus 2.0 Array), GPL570 [(HG-U133_Plus_2) Affymetrix Human Genome U133 Plus 2.0 Array], GPL6244 [(HuGene-1_0-st) Affymetrix Human Gene 1.0 ST Array], respectively, and the features applied for validation were derived from the same method used in the derivation cohort (DEGs and CIBERSORTx).

## Results

### Composition of Immune Cells in CD and Normal Tissues

**FIGURE 1:**
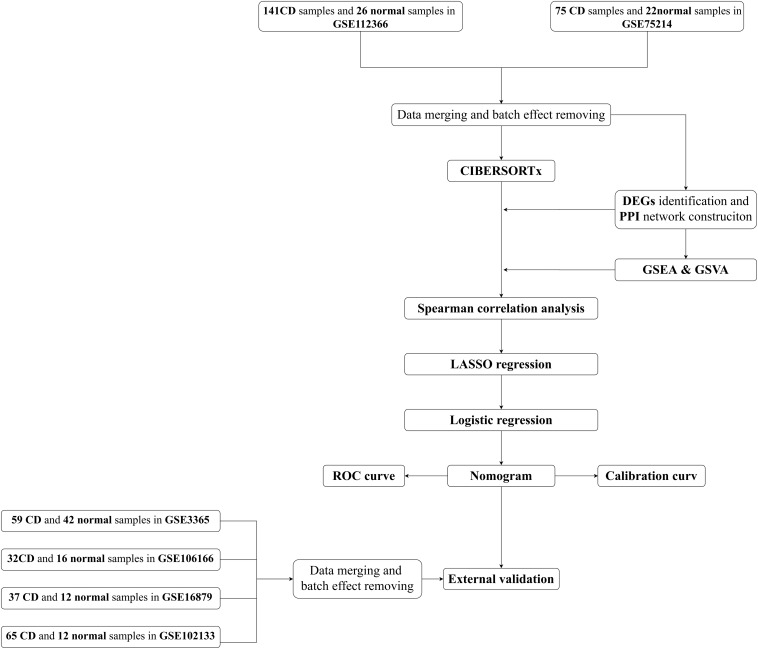
Flow chart of the analyses used in this study.

**FIGURE 2:**
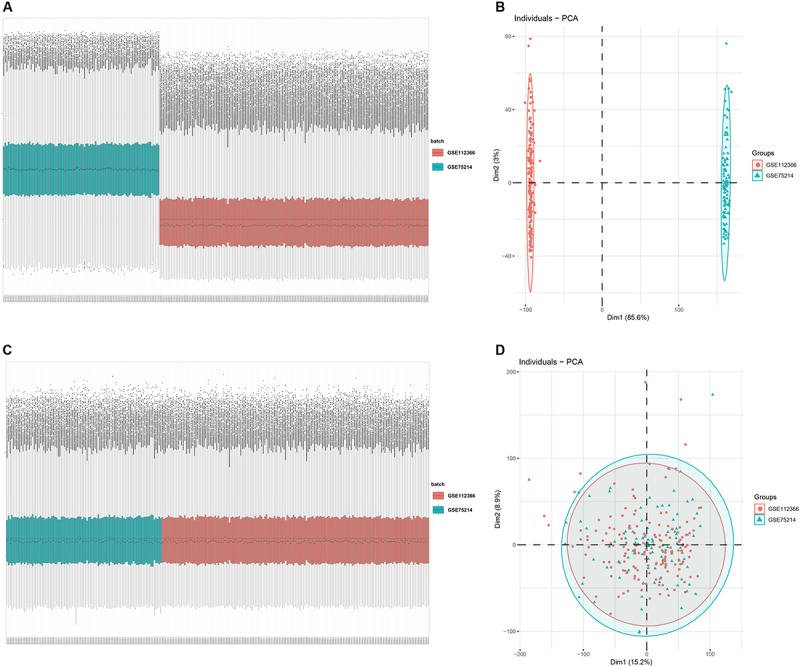
Data preprocessing of the derivation cohort. Box plot and principal component analysis showing the overall profiles of GSE112366 and GSE75214 **(A,B)** before and **(C,D)** after normalization. The results confirmed removal of the batch effect PCA.

**FIGURE 3:**
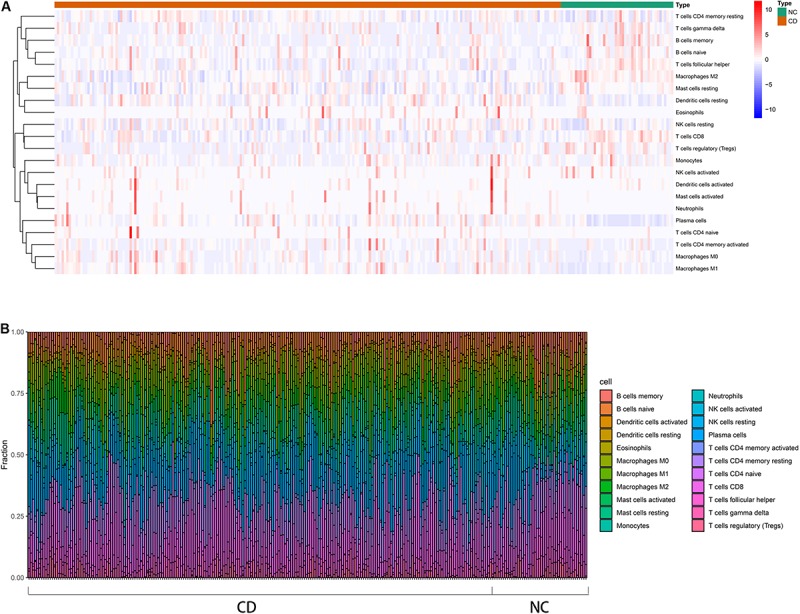
Distribution of immune cells between CD patients and healthy controls using CIBERSORTx for all eligible samples in the derivation cohort. **(A)** Heatmap of the normalized absolute abundance for each cell type in individual samples. **(B)** Barplot of different the fractions of immune cells in individual samples.

**FIGURE 4:**
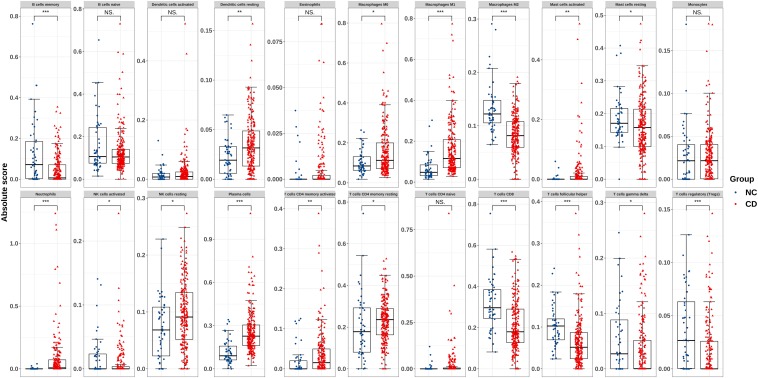
Boxplot of comparisons of immune-cell proportion between CD patients and healthy controls in the derivation cohort. The red boxplot represents CD patients, and the blue boxplot represents healthy controls. The fraction of resting DCs, macrophages (M0 and M1), activated mast cells, and neutrophils were higher in CD patients, whereas the fraction of M2 macrophages, resting mast cells, and γδT cells were lower in CD patients. The number of resting NK cells, plasma cells, CD4 memory T cells (activated and resting) were elevated in CD, whereas that of activated NK cells, memory B cells, CD8 T cells, follicular helper T cells, and Tregs was lower. *p < 0.05, **p < 0.01, ***p < 0.001.

An overview of the workflow is shown in Figure 1 and the R code is available in Supplementary Data Sheet S1. The derivation cohort contained 216 CD tissue samples and 48 normal bowel tissue samples. Data before and after normalization were visualized in a boxplot and examined by principal component analysis (PCA), and suggested that the batch effect of merged data was successfully removed (Figure 2). After algorithm execution, all samples were enrolled with a CIBERSORTx output with a *P* < 0.05 according to a previously defined threshold (Supplementary Tables S1, S2). The distribution of immune cells is shown in a heatmap (Figure 3A) and barplot (Figure 3B), indicating that many were significantly altered among groups. The Wilcoxon test revealed that the innate immune system showed a higher fraction of resting DCs, macrophages (M0 and M1), activated mast cells, and neutrophils in CD and a lower fraction of M2 macrophages, resting mast cells, and γδT cells in CD patients (*P* < 0.05), whereas in the adaptive immune system, resting NK cells, plasma cells, and CD4 memory T cells (activated and resting) were more prominent in CD, whereas a decreased proportion of activated NK cells, memory B cells, CD8 T cells, follicular helper T cells, and regulatory T cells (Tregs) were observed (*P* < 0.05) (Figure 4).

### Functional Enrichment Analysis of DEGs and Identification of Hub Genes

**FIGURE 5:**
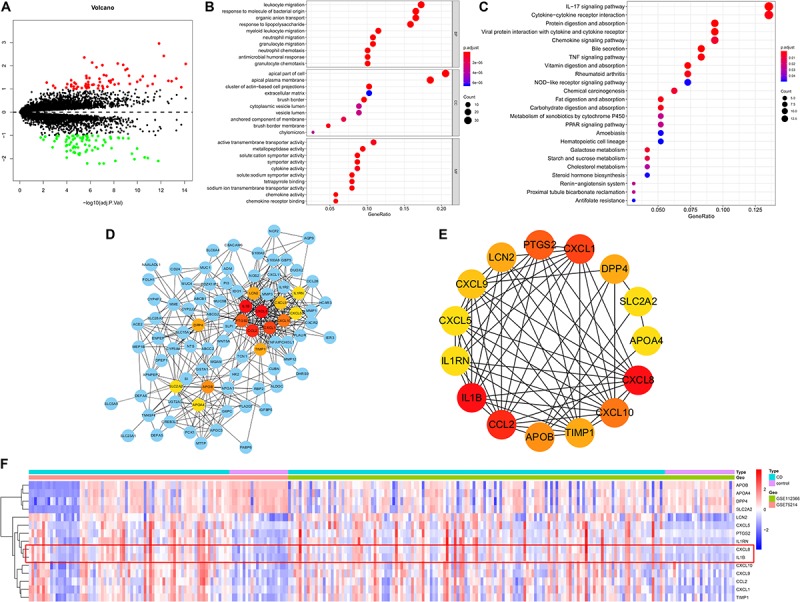
DEGs identified in the derivation cohort. **(A)** Volcano plot of DEGs. Red dots represent relatively upregulated genes and green dots represent downregulated genes. Black dots represent genes showing no significant alteration. There were 150 DEGs, including 70 upregulated and 80 downregulated genes among 17,689 genes. Bubble plot of **(B)** Gene Ontology and **(C)** KEGG enrichment analyses. **(D)** Cytoscape software was used for the analysis. **(E)** Hub genes with the top 15 degrees of interaction were identified using Cytohubba plugin. **(F)** Heatmap of the hub genes. BP, biological process; CC, cellular component; MF, molecular function.

Based on predefined cut-off criteria, we obtained 150 DEGs, including 70 upregulated genes and 80 downregulated genes, from a total of 17,689 genes in derivation cohort (Figure 5A and Supplementary Figure S1). GO enrichment analysis revealed that DEGs were primarily related to the immune response (e.g., leukocyte migration, response to molecules of bacterial origin, organic anion transport, response to lipopolysaccharide, neutrophil migration, and cytokine activity) (Figure 5B). KEGG enrichment analysis demonstrated that DEGs were mainly enriched in pathways related to immunity, such as interleukin (IL)-17 signaling, nucleotide-binding oligomerization domain (NOD)-like receptor signaling, tumor necrosis factor (TNF) signaling, chemokine signaling (Figure 5C). The interactions among 150 DEGs were visualized in the PPI network, which was constructed using an online database (version: 11.0). We identified 145 nodes and 536 edges among the DEGs and used Cytoscape for visualization (Figure 5D). Genes with the top 15 scores based on Cytohubba analysis were identified as hub genes (Figure 5E and Table 1) and the expression distribution of the hub genes is shown as a heatmap (Figure 5F). Among the identified genes, both C-X-C chemokine ligand 8 (CXCL8) and IL-1B showed the highest degrees of interaction; therefore, we considered these as potentially crucial genes in CD pathogenesis.

**TABLE 1:** The expression analysis of the top 15 hub genes with the highest interaction degree.

**Gene symbol**	**LogFC**	***P*. Value**	**Adj. *P*. Value**	**Degree**
CXCL8	1.573168	6.38E-10	4.41E-08	34
IL1B	2.037914	1.02E-11	1.60E-09	34
CCL2	1.189715	4.87E-08	1.49E-06	29
CXCL11	1.216482	1.01E-06	1.70E-05	28
PTGS2	1.105027	1.64E-06	2.57E-05	25
CXCL10	1.185171	3.02E-06	4.20E-05	22
APOB	−1.95788	9.08E-06	0.000103	20
TIMP1	1.178994	1.82E-08	6.87E-07	19
LCN2	2.422802	4.46E-16	5.63E-13	19
DPP4	−1.02179	2.30E-06	3.37E-05	19
CXCL9	1.274086	1.43E-06	2.28E-05	18
SLC2A2	−1.27104	1.10E-05	0.00012	17
CXCL5	1.115601	3.04E-06	4.22E-05	17
APOA4	−1.45209	1.03E-05	0.000114	17
IL1RN	1.191117	1.32E-08	5.18E-07	17

### Identification of CD-Associated Pathways via GSEA and GSVA

**FIGURE 6:**
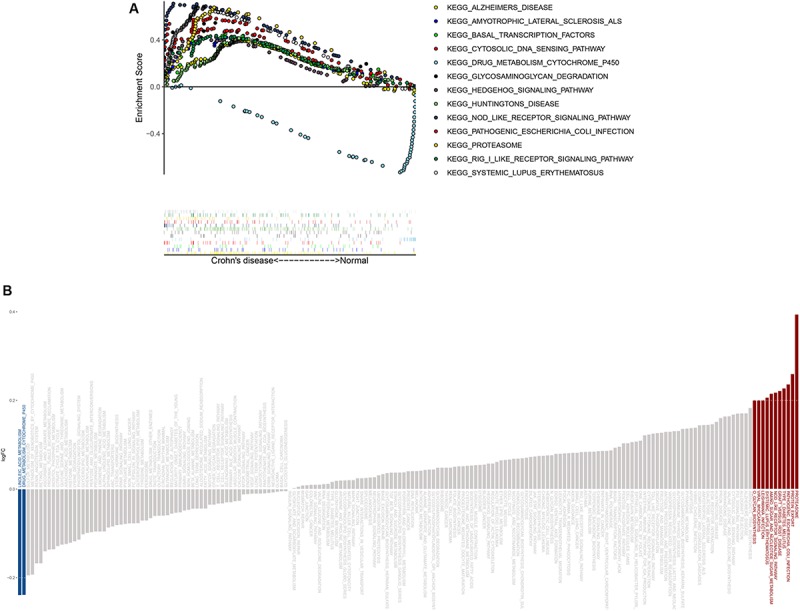
GSEA and GSVA results from the derivation cohort. **(A)** GSEA revealed that most of the enriched pathways (*P* < 0.05) correlated with CD. **(B)** GSVA showed that most of the significantly altered pathways were activated in CD.

GSEA results showed that genes in the disease group were significant highly enriched in 12 pathways, with only one pathway enriched in the healthy group (*P* < 0.05) (Figure 6A and Supplementary Figure S2). Similarly, 10 pathways were significantly activated in CD, whereas two were inhibited according to GSVA results (Figure 6B). Five pathways (“Proteasome,” “Pathogenic Escherichia coli infection,” “NOD-like receptor signaling pathway,” “Drug metabolism cytochrome p450,” and “Systemic lupus erythematosus”) overlapped in both GSEA and GSVA results and were chosen for further correlation analysis (Table 2). However, only the NOD-like receptor signaling pathway was identified in the former KEGG pathway enrichment results for the DEGs.

**TABLE 2:** The pathways overlapped in results of GSEA and GSVA.

**Pathway**	**GSEA**		**GSVA**	
	
	**NES**	***P*. Val**	**logFC**	**Adj. P. Val**
KEGG_PROTEASOME	1.84	0.010	0.3942	6.21E-08
KEGG_PATHOGENIC_ESCHE				
RICHIA_COLI_INFECTION	1.43	0.049	0.236553	1.22E-05
KEGG_NOD_LIKE_RECEPTOR_				
SIGNALING_PATHWAY	1.62	0.004	0.217978	1.76E-05
KEGG_DRUG_METABOLISM_				
CYTOCHROME_P450	−1.41	0.044	−0.23917	0.000208
KEGG_SYSTEMIC_LUPUS_				
ERYTHEMATOSUS	1.60	0.013	0.206671	0.001946

### Co-expression Analysis and Variable Selection

**FIGURE 7:**
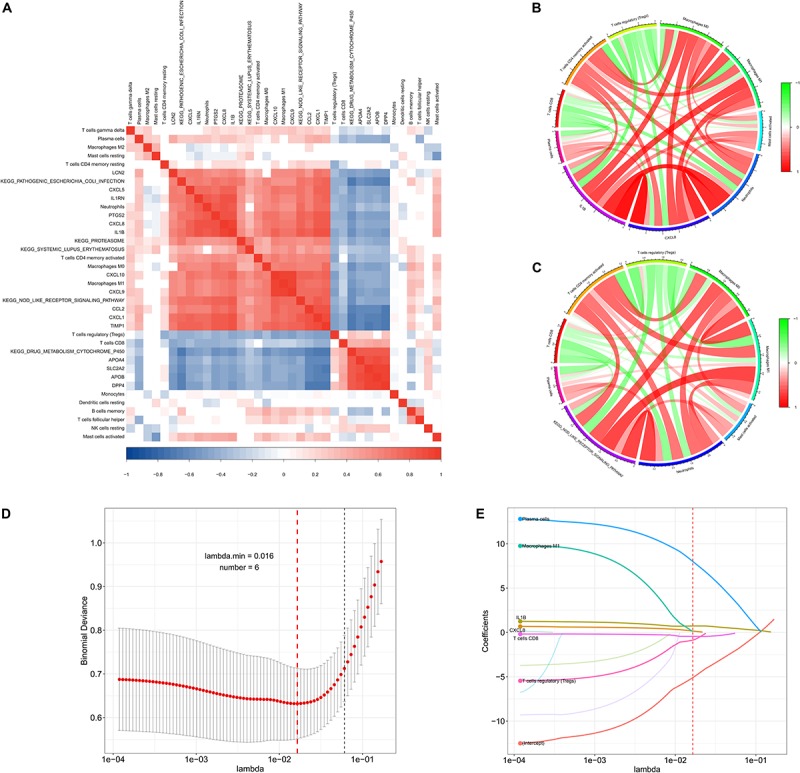
Co-expression patterns and LASSO regression of the derivation cohort. **(A)** Correlation heatmap showing gene co-expression patterns among significantly altered immune cells, hub genes, and pathways. **(B,C)** Circos plot showing the relationships between immune cells, CXCL8 and IL1-B levels, and the NOD-like signaling pathway (| correlation coefficient| > 0.3). **(D,E)** LASSO regression analysis identified six factors with cross-validation performed to prevent overfitting.

To identify co-expression patterns among significantly altered immune cells, hub genes, and signaling pathways, we performed Spearman correlation analysis to evaluate possible relationships (Figure 7A and Supplementary Tables S3, S4). The results showed that γδT cells, plasma cells, neutrophils, activated CD4 memory T cells, macrophages (M0 and M1) and activated mast cells showed a positive correlation with most of the genes and signaling pathways, whereas Tregs and CD8 T cells showed a negative correlation with the same. To explore potential regulatory networks, we identified the immune cells with a | correlation coefficient| > 0.3 for CXCL8 and IL-1B levels that showing the highest degrees of interaction in hub genes (Figure 7B). Theses included plasma cells, CD8 T cells, activated CD4 memory T cells, Tregs, macrophages (M0 and M1), activated master cell, and neutrophils, and interestingly, these cells were also correlated with the NOD-like receptor signaling pathway (Figure 7C). These 11 features, including levels of CXCL8 and IL-1B, the NOD-like receptor signaling pathway, and their associated immune cells, were integrated into the LASSO regression model, resulting in six features with non-zero coefficients used for further nomogram construction (Figures 7D,E).

### Construction of the Diagnostic Prediction Model

**FIGURE 8:**
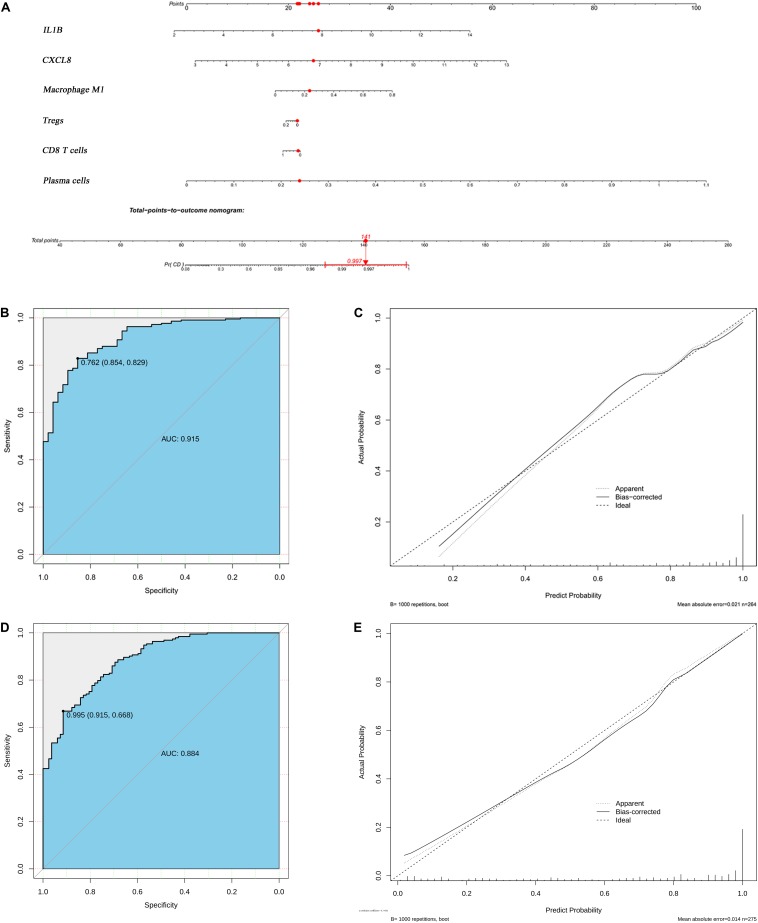
Logistic regression analyses of both the derivation and validation cohorts. **(A)** Nomogram predicting CD probability. **(B)** ROC curve and **(C)** calibration curve of the model from derivation cohort. **(D)** ROC curve and **(E)** the calibration curve of the model from validation cohort.

CXCL8 and IL-1B levels, M1 macrophages, Tregs, CD8 T cells, and plasma cells were incorporated into a multivariable logistic regression model to build a diagnostic prediction model for CD and presented as a nomogram (Figure 8A). The area under the ROC analysis for this model is 0.915 (Figure 8B), and the calibration curve showed that the model agreed well between the actual and predicted probability of CD occurrence (Figure 8B). The C-index of the nomogram for predicting CD occurrence was 0.915 [95% confidence interval (CI): 0.875–0.955]. Moreover, the Pearson residuals plot showed that the relationship between residuals and predictors was nearly linear without curvature (Supplementary Figure S6A).

### Validation of the Diagnostic Model

The validation cohort contained a total of 193 CD tissue samples and 83 normal bowel tissue samples. The batch effect of the merged data was successfully removed and visualized using the same method used for the derivation cohort (Supplementary Figure S3), and DEG and CIBERSORTx results were similar to those of the derivation cohort (Supplementary Figures S4, S5). The area under the ROC analysis for this model was 0.884 (Figure 8D), and the calibration curve showed that the model in the validation cohort also agreed well between the probabilities of actual and predicted CD occurrence (Figure 8E). The C-index of the validation model predicting CD occurrence was 0.884 (95% confidence interval: 0.843–0.924). Furthermore, the trend of Pearson residuals plot was similar to that from derivation cohort (Supplementary Figure S6B).

## Discussion

CD is one of the major types of IBDs and a chronic relapsing inflammatory process that mainly affects the gastrointestinal tract (Baumgart and Carding, 2007), with dysregulated immune-cell trafficking in the intestine representing the predominant event in CD pathogenesis (Zundler et al., 2019). Although previous studies have attempted to elucidate the precise pathological process, few reports have provided an overview of immune-cell alterations in CD. Therefore, we employed bioinformatics analyses as a viable strategy to investigate the profile of immune cells to offer insight into the collective regulatory mechanism of CD.

We used CIBERSORTx to estimate the fraction of 22 immune cells from the innate and adaptive immune systems in CD patients and healthy controls, and established regulatory co-expression regulatory patterns based on correlation analyses between immune cells, genes, and signaling pathways. Moreover, we generated a predictive diagnostic nomogram revealing good performance using both derivation and validation cohort.

Our diagnostic model showed that CXCL8 and IL-1B represented hub genes with the highest degree of interaction, suggesting their important role in CD pathogenesis. A well-known function of CXCL8 is neutrophils activation and attraction, based on their recruitment to inflamed intestinal mucosa during the early stage of the inflammatory response (Kolaczkowska and Kubes, 2013; Russo et al., 2014). Our analysis identified a higher fraction of neutrophils in CD relative to that in normal tissue along with the highest correlation with CXCL8 levels. These results suggested that neutrophils are essential for CD progression; however, whether its role is pathological or beneficial remains controversial. Recently, single-cell analysis revealed that neutrophils recruitment and activation was correlated with clinical disease severity in CD (Therrien et al., 2019). However, Zhou et al. (2018) demonstrated that the CD177^+^ neutrophils showed a protective effect in IBD through increased anti-bacterial activity and IL-22 production. Therefore, this discrepancy might be due to difference in neutrophils subsets and the phases in which they appear during inflammation.

IL-1β is an important pro-inflammatory cytokine that promotes the inflammatory process and coordinates autoimmune responses (Ligumsky et al., 1990). IL-1β is correlated with disease severity, implying essential in pathogenesis of IBD (McAlindon et al., 1998; Young et al., 2017). Stimulated by microbe-associated molecular patterns (MAMPs), IL-1β from macrophages, DCs, and epithelial cells induces the development of CD4^+^ T cells (de Souza and Fiocchi, 2016). Moreover, both M1 (pro-inflammatory phenotype) and M2 (pro-resolving phenotype) macrophages are essential for intestinal immune homeostasis (Murray et al., 2014) with M1 macrophages essential for the antibacterial response that involves proinflammatory cytokines production (i.e., IL-1β) mediate acute inflammation (Wynn et al., 2013). Therefore, intestinal macrophages dysregulation possibly underlies chronic inflammation associated with IBD due to a lower tolerance of bacteria and food antigens (de Souza and Fiocchi, 2016). The present study demonstrated an increased proportion of M1 macrophages in CD patients, whereas that of M2 macrophages was lowered. Moreover, we found an increased fraction of CD4^+^ memory T cells in CD patients that was positively correlated with IL-1B levels; however, due to limitations with CIBERSORTx analysis, we were unable to investigate subsets of CD4^+^ T cell (e.g., CD-specific changes in proportions of T-helper cell).

However, CIBERSORTx analysis offered an overview of Treg and follicular help T cells (Tfh) status as subsets of CD4 + T cells. The results showed that the fraction of theses cells decreased in CD patients, and that Treg level was an important predictor in the diagnostic model. However, a previous study reported an increased percentage of Tregs as part of CD diagnosis, as well as during the active phase of the disease (Smids et al., 2018). Tregs generally suppress IL-1B production and inhibit effector T cell proliferation, whereas some studies reported that these function are impaired in CD (Maloy and Powrie, 2011; Cook et al., 2019). This suggests that Treg status in CD remains controversial and might depend on inflammatory stage. Another important type of T cell in our diagnostic model was the CD8^+^ T cell, which showed a decreased proportion in CD patients. Similar to our results, a previous study reported a decreased response to commensal microbiota by CD8^+^ T cells in IBD (Noble et al., 2019). Because the antigen-presenting function of DCs is critical for the CD8^+^ T cell response, the higher percentage of inactivated DCs identified in our analysis might provide a possible explanation. Moreover, another study indicated that polymorphisms of NOD2, which has been widely studied for its strong association with CD, contribute to dysregulated cross-presentation of DCs from CD, ultimately leading to impaired CD8^+^ T cell response (Corridoni et al., 2019). Surprisingly, our results also revealed that the activated NOD-like receptor signaling pathway plays a key role in CD occurrence and is negatively correlated with the CD8^+^ T cell levels. Although the NOD-like receptors (NLRs) are essential for the anti-microbial response, defective autophagy due to NOD2 polymorphisms accounts for attenuated bacterial clearance and activation of the downstream nuclear factor kappaB (NF-κB) pathway activation (Travassos et al., 2010; Plantinga et al., 2011).

Notably, reduced CD8 + T cell levels might lead to a skewing toward a humoral immune response, where antibodies are produced by activated B cells (Noble et al., 2019). Despite the lack of studies targeting the role of B cells in CD, one study identified B cell disruption in CD, including increased levels of plasma cells in lamina propria and altered production of a subclass of antibodies (Brandtzaeg et al., 2006). Consistent with these findings, our results showed a higher percentage of plasma cells in CD patients relative to healthy controls, with this contributing to the predictions in the nomogram. Additionally, unlike plasma cells, the fraction of naïve and memory B cells was reduced; however, the exact pathogenic role of B cell distribution remains unclear. A previous study reported that an antibody targeting tumor necrosis factor- α normalized levels of traditional B cells, suggesting the tumor necrosis factor- α as a potential biomarker for treatment monitoring (Timmermans et al., 2016). Moreover, we found that the reduced levels of γδ-T cells in CD tissue comprised only a small proportion of resident lymphocytes. Although previous studies observed similar decreases in the intestinal mucosa, the plasticity of γδ T cells make it difficult to elucidate the exact pathogenic role (Catalan-Serra et al., 2017); therefore, further research is necessary to elucidate the function of γδ T cells in CD.

Our study has some limitations. First, due to the lack of clinical information, including disease phenotype, disease activity status, and CD Activity Index score, associations between immune cells and disease severity could not be well estimated. Second, the score of each factor in the nomogram was derived from gene expression data and an absolute value produced by CIBERSORTx; therefore, our model emphasized the factor itself rather than the value, and a normalization method should be developed for further application of the nomogram. Third, our control included normal intestine tissue. Some diseases that show confusing similarities with CD, including intestinal tuberculosis or Behcet’s disease, need to be used for comparison; however, to the best of our knowledge, this was the first study using the CIBERSORTx algorithm to identify the proportion of immune cells in CD and provide novel biomarkers for diagnostic prediction. In future work, molecular biological experiments and/or flow cytometry analyses need to be performed to validate these findings, and another external validation based on a larger sample should be conducted.

## Conclusion

These data offer insight into the landscape of immune cells associated with CD and provide information for a auxiliary diagnosis based on co-expression patterns within an immune-cell cohort specific to CD. The findings demonstrated the cellular and molecular heterogeneity in the disease, and are consistent with previous studies. These results provide novel insight into the cellular and molecular mechanisms underlying CD and facilitate accurate diagnosis of the likelihood of CD.

## Data Availability Statement

Publicly available datasets were analyzed in this study. The datasets (GSE112366, GSE75214, GSE3365, GSE10616, GSE16879, and GSE102133) provided by Gene Expression Omnibus can be found here: GEO, https://www.ncbi.nlm.nih.gov/geo/.

## AutHor Contributions

HC designed the experiments, analyzed the data, and wrote the manuscript. CC, XY, WX, MY, and QL designed the experiments and analyzed the data. ZS revised the manuscript. LY devised the concept, designed the research, supervised the study, and wrote the manuscript. All authors read and approved the final manuscript.

## Conflict of Interest

The authors declare that the research was conducted in the absence of any commercial or financial relationships that could be construed as a potential conflict of interest.

## Footnotes

1 https://cibersortx.stanford.edu/

2 http://string-db.org; version11.0

3 http://software.broadinstitute.org/gsea/index.jsp

## Supplementary Material

FIGURE S1 The heatmap of DEGs from derivation cohort.

FIGURE S2 The heatmap of GSVA results from derivation cohort.

FIGURE S3 Data preprocessing of the validation cohort. Box plot and principal component analysis showing the overall profiles of GSE3365, GSE10616, GSE16879, and GSE102133 **(A,B)** before and **(C,D)** after normalization. The results confirmed removal of the batch effect.

FIGURE S4 The heatmap of DEGs from validation cohort.

FIGURE S5 Boxplot of comparisons of immune-cell proportion between CD patients and healthy controls in the validation cohort.

FIGURE S6 Pearson plot of the model from derivation cohort **(A)** and validation cohort **(B)**.

TABLE S1 The results of CIBERSORTx in Crohn’s disease.

TABLE S2 The results of CIBERSORTx in normal control.

TABLE S3 The correlation coefficients of co-expression patterns in derivation cohort.

TABLE S4 The correlation *P*-value of co-expression patterns in derivation cohort.

DATA SHEET S1 The R code used in our study.
