# Supplementary figures and images for: Identification of Immune Cell Landscape and Construction of a Novel Diagnostic Nomogram for Crohn’s Disease
Source: Front Genet. 2020 Apr 29;11:423. doi: 10.3389/fgene.2020.00423 (PMC7212409; doi:10.3389/fgene.2020.00423)

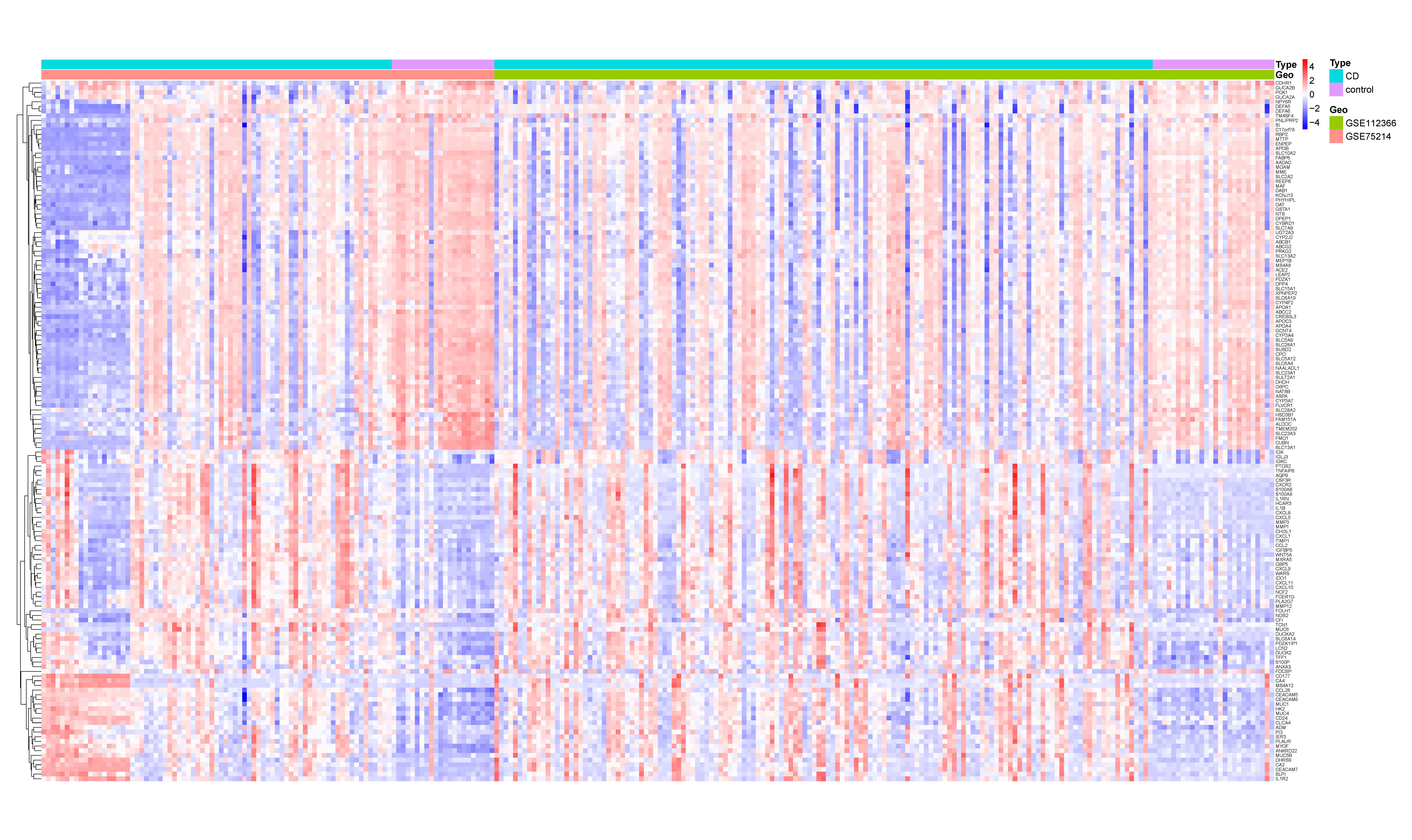

Supplement: FIGURE S1 — The heatmap of DEGs from derivation cohort. [file Image_1.jpg]

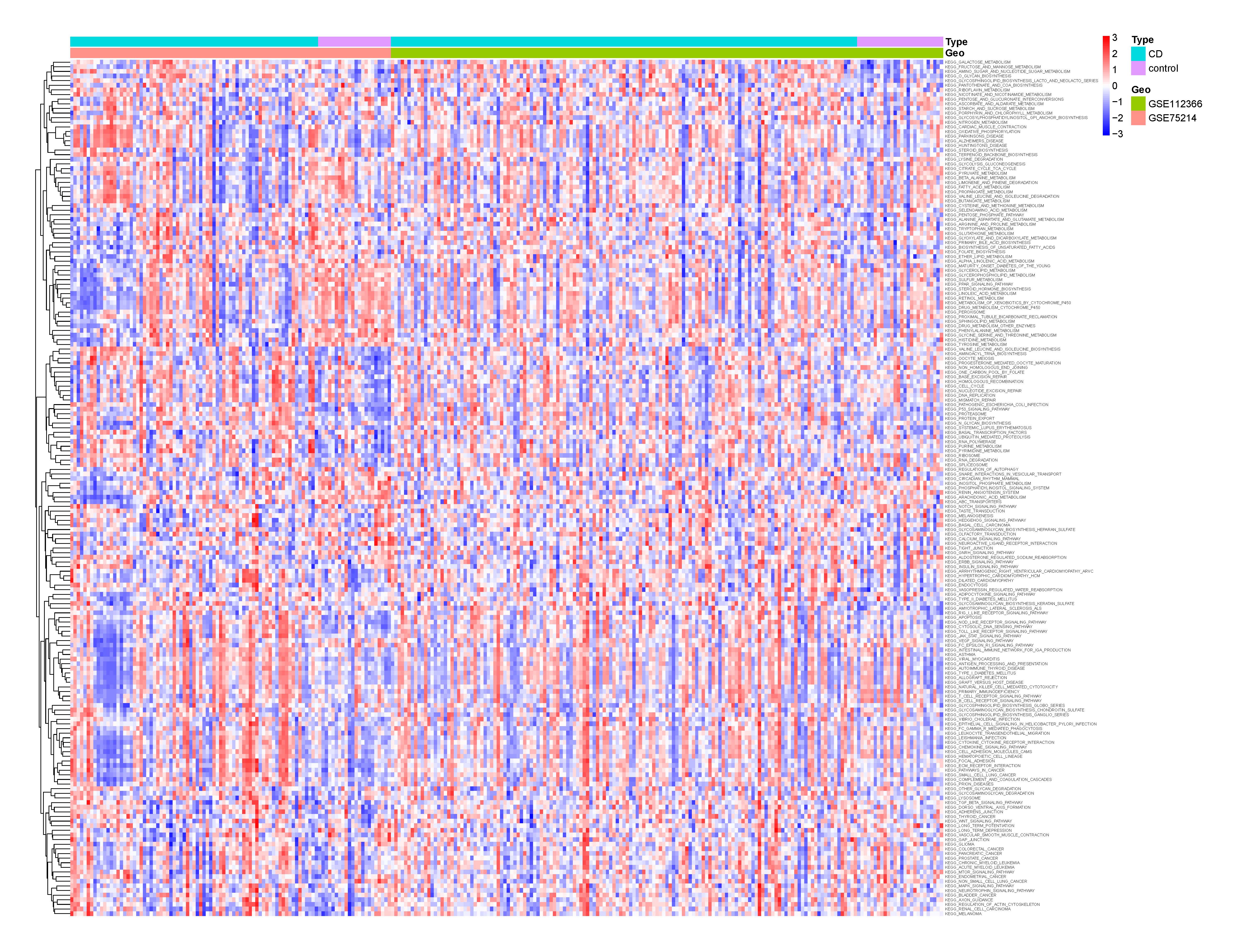

Supplement: FIGURE S2 — The heatmap of GSVA results from derivation cohort. [file Image_2.jpg]

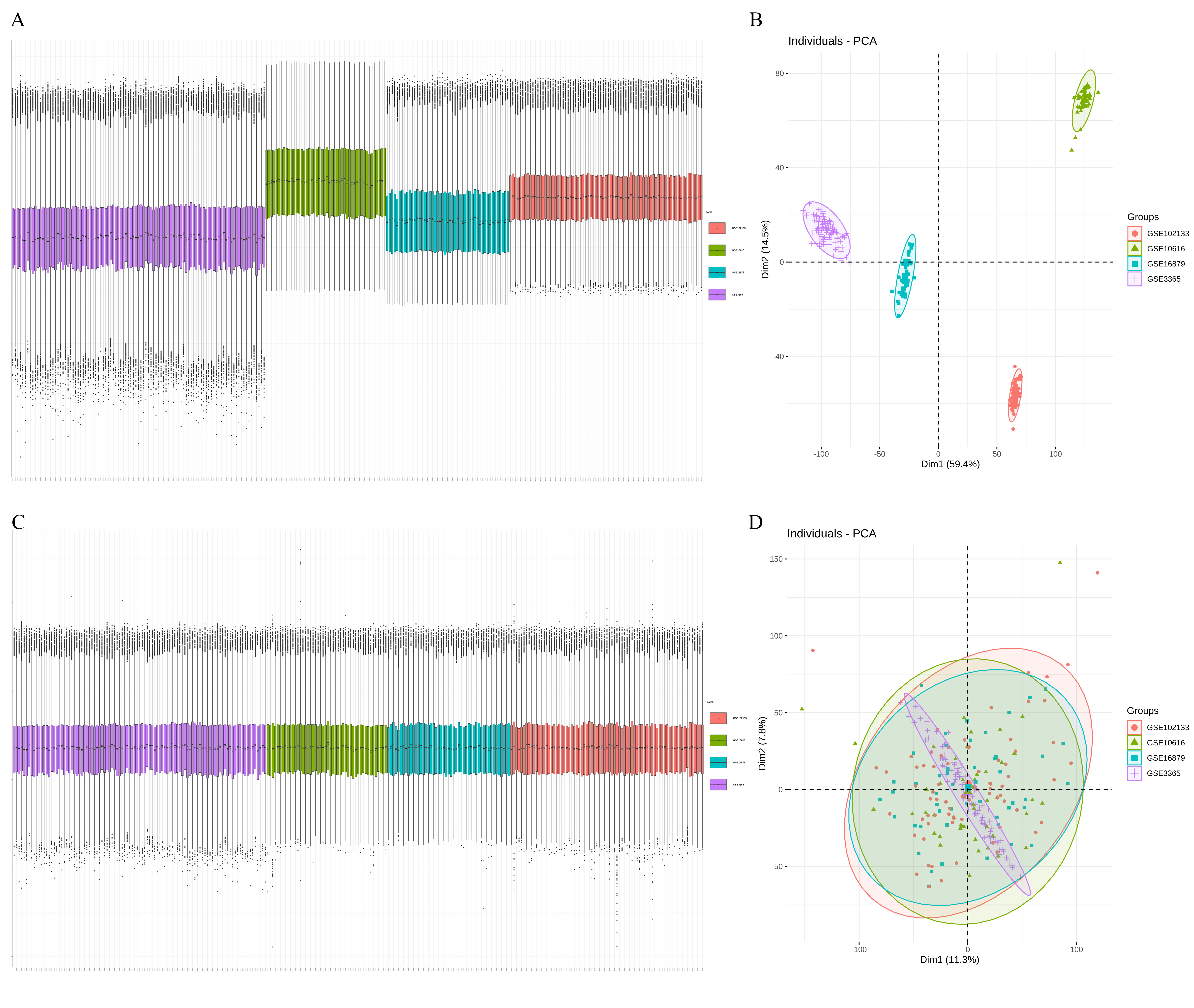

Supplement: FIGURE S3 — Data preprocessing of the validation cohort. Box plot and principal component analysis showing the overall profiles of GSE3365, GSE10616, GSE16879, and GSE102133 (A,B) before and (C,D) after normalization. The results confirmed removal of the batch effect. [file Image_3.jpg]

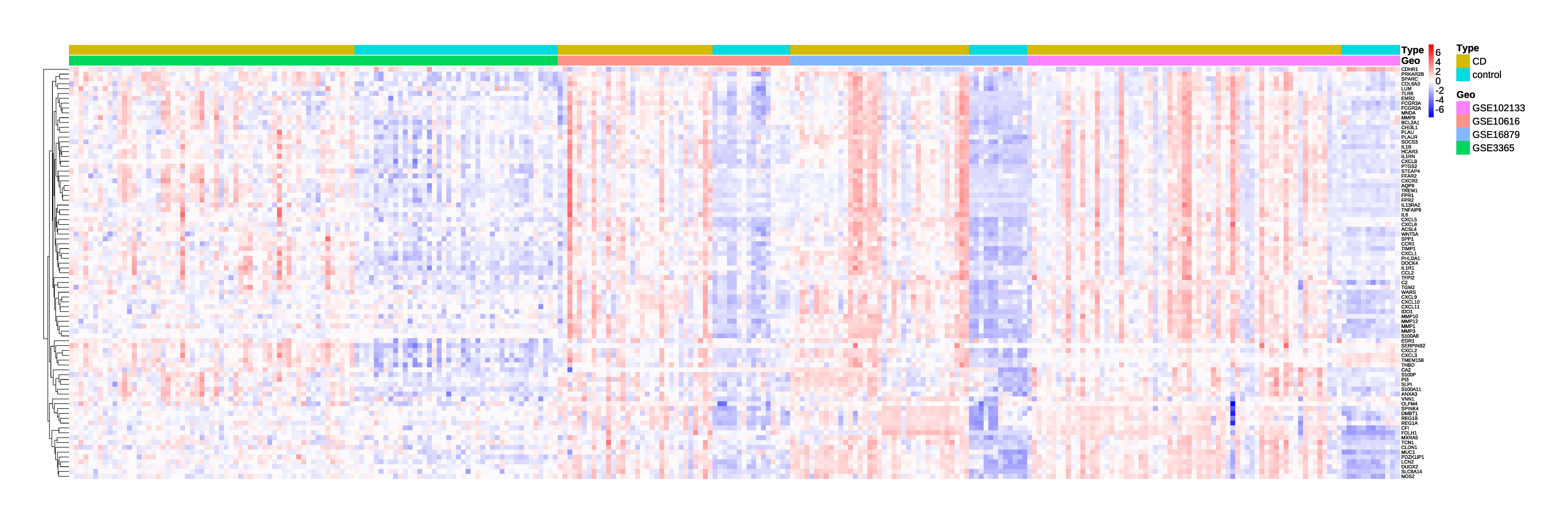

Supplement: FIGURE S4 — The heatmap of DEGs from validation cohort. [file Image_4.jpg]

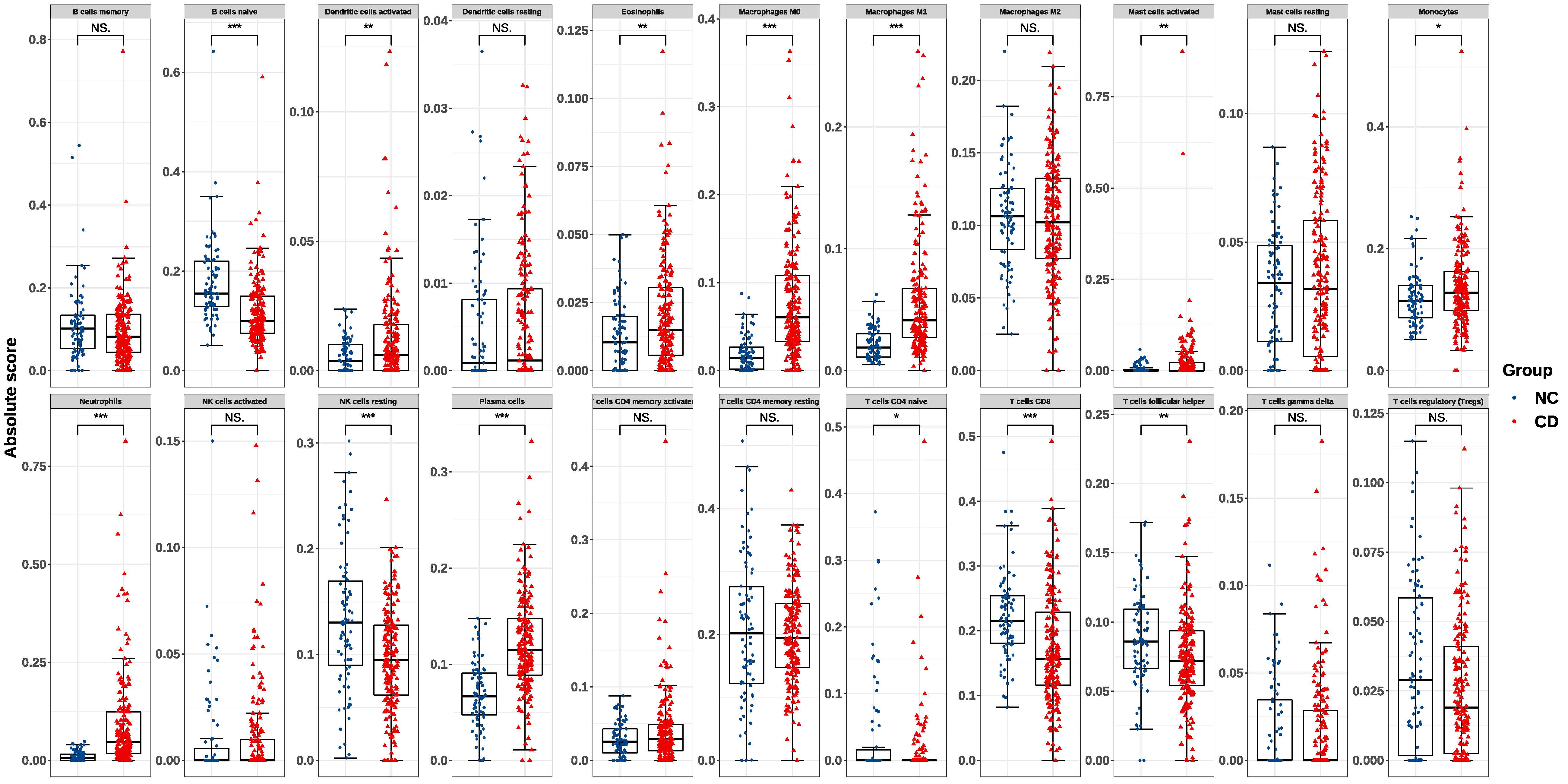

Supplement: FIGURE S5 — Boxplot of comparisons of immune-cell proportion between CD patients and healthy controls in the validation cohort. [file Image_5.jpg]

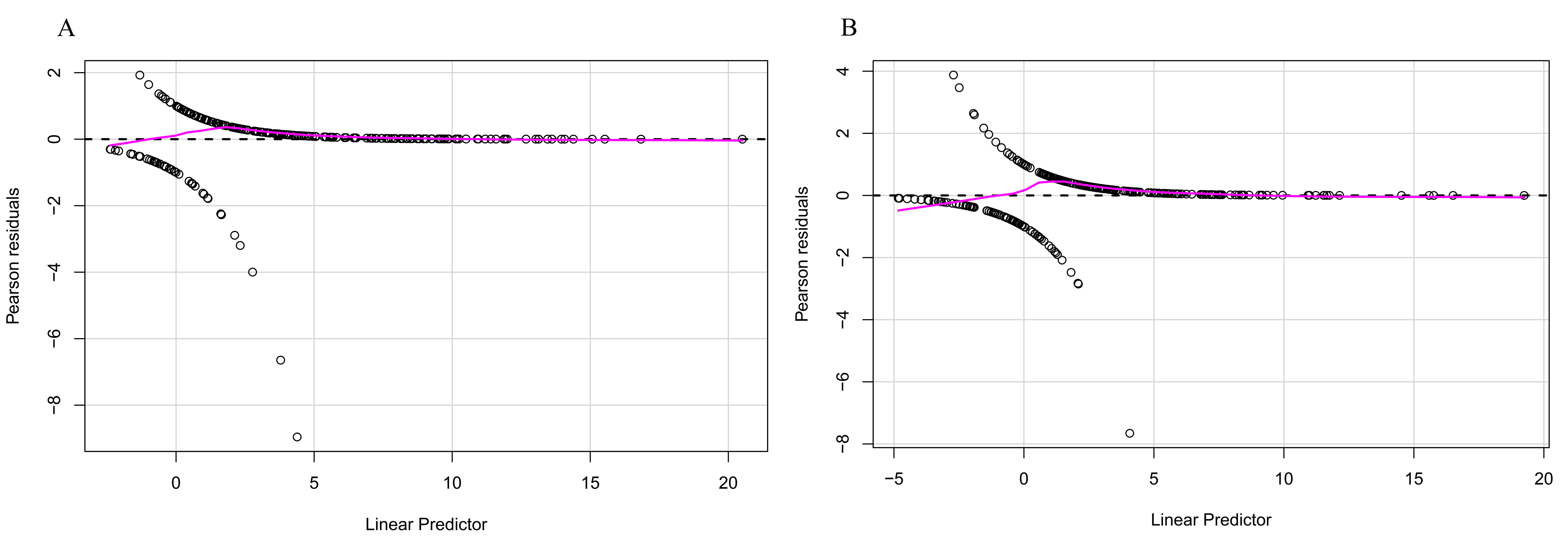

Supplement: FIGURE S6 — Pearson plot of the model from derivation cohort (A) and validation cohort (B). [file Image_6.jpg]
